# Supplementary material for: From sequence to enzyme mechanism using multi-label machine learning
Source: BMC Bioinformatics. 2014 May 19;15:150. doi: 10.1186/1471-2105-15-150 (PMC4229970; doi:10.1186/1471-2105-15-150)
Supplement: Additional file 2 — Java code of ml2db. Additional file ml2db_code.tar.gz contains the Java source code to run the multi-label machine learning experiments and save the results to database. The code’s Javadoc is included. [file 1471-2105-15-150-S2.zip › additional file 2/ml2db/ml2db/doc/mulan/classifier/meta/package-frame.html]

mulan.classifier.meta


# mulan.classifier.meta

## Classes

- ClusteringBased
- ConstrainedKMeans
- ConstrainedKMeans.bucketInstance
- EnsembleOfSubsetLearners
- HierarchyBuilder
- HMC
- HMCNode
- HOMER
- MultiLabelMetaLearner
- RAkEL
- RAkELd
- SubsetLearner

## Enums

- HierarchyBuilder.Method
